# Group-Based Metacognitive Reflection and Insight Therapy (MERITg) and Its Relationship to Recovery-Oriented Beliefs in Serious Mental Illness

**DOI:** 10.3390/bs14070520

**Published:** 2024-06-22

**Authors:** Christie W. Musket, Joshua Bullock, Joanna M. Fiszdon, Meaghan Stacy, Steve Martino, Alison James, Paul H. Lysaker, Ashley M. Schnakenberg Martin

**Affiliations:** 1 Psychology Service, VA Connecticut Healthcare System, West Haven, CT 06516, USA; christie.musket@yale.edu (C.W.M.); meaghan.stacy@yale.edu (M.S.); steve.martino@yale.edu (S.M.); 2 Department of Psychiatry, Yale University School of Medicine, New Haven, CT 06511, USA; 3 Psychology Service, VA Maryland Healthcare System, Baltimore, MD 21201, USA; alison.james@va.gov; 4 Psychology Service, Roudebush Veteran Affairs Medical Center, Indianapolis, IN 46202, USA; 5 Department of Psychiatry, Indiana University School of Medicine, Indianapolis, IN 46202, USA

**Keywords:** recovery-oriented beliefs, serious mental illness, MERIT, MERITg, metacognition, group psychotherapy

## Abstract

Group-based Metacognitive Reflection and Insight Therapy (MERITg) is the group application of Metacognitive Reflection and Insight Therapy (MERIT), an evidence-based, integrative, recovery-oriented intervention to enhance insight and understanding of oneself and others in individuals with serious mental illness (SMI). MERITg may offer therapeutic interactions between participants that uniquely support recovery. The goal of the current study was to examine the relationship between MERITg participation and recovery-oriented beliefs. Thirty-one participants (outpatient = 21; inpatient = 10) in SMI treatment programs participated in MERITg as an adjunctive treatment. A short form of the Maryland Assessment of Recovery in Serious Mental Illness (MARS-12) was used to assess recovery-oriented beliefs before and after group participation. Recovery-oriented beliefs significantly improved in the outpatient MERITg group but not in the inpatient group, and change in recovery-oriented beliefs was positively correlated with the total number of groups attended. These findings suggest the promise of MERITg for enhancing recovery-oriented beliefs. The potential role of treatment setting is discussed.

## 1. Introduction

Serious mental illness (SMI) is a broad term that can refer to a wide range of psychiatric diagnoses and experiences. At its core, it signifies the presence of both a psychiatric diagnosis and significant functional impairment, and it most frequently includes psychotic (e.g., schizophrenia) and mood (e.g., bipolar disorder and major depression) disorders [1]. Historically, providers tended to focus narrowly on symptom reduction as the primary goal of treatment for individuals with SMIs, and attitudes towards treatment response were often pessimistic. The prevailing wisdom was that individuals with such conditions could hope for stability and very little else. In recent years, however, the recovery movement has cultivated a more optimistic mindset and widened the scope of what successfully living with these conditions might mean. Rather than focusing exclusively on symptom reduction or on goals set by professionals, recovery-oriented care focuses on how individuals can find meaning and purpose that is unique to them and led by them, in a way that is not necessarily focused on the potential confines or difficulties presented by psychiatric symptoms [2,3,4]. 

The concept of recovery is highly individualized and inherently means different things to different individuals; however, significant effort has been made to clearly define and operationalize recovery (e.g., [5]). Common themes that have emerged as essential to recovery have included self-direction or self-empowerment, hope, responsibility, a strengths-focus, respect, and acceptance of the non-linearity of experiences [6]. It is therefore important to actively consider and monitor recovery-oriented beliefs throughout clinical interventions for individuals with SMI. While there are many different strengths and skills that factor into an individual’s trajectory in recovery, one particularly important domain is metacognition, or “thinking about thinking”. Metacognition can be thought of as a spectrum of mental activities and includes noticing and observing one’s own and others’ mental states, and integrating those thoughts and beliefs into a complex representation of the self and others [7]. Given that recovery involves cultivating a deeper understanding of one’s sense of self, desire, and agency in life, metacognition is an invaluable part of the recovery process. A robust body of research has found that individuals with SMIs, like schizophrenia, often experience profound difficulties with insight and metacognition [7,8], and that such difficulties have been consistently linked with poorer outcomes, such as increased symptom severity and lower psychosocial functioning [9]. Importantly, metacognition is a skill that can be cultivated and can improve with practice and intervention. Metacognitive Reflection and Insight Therapy (MERIT) was developed to specifically address metacognition in the service of helping individuals reach recovery-oriented goals [10]. 

MERIT strongly aligns the values of the recovery movement. MERIT was originally conceptualized as a one-on-one intervention to support the collaborative efforts of clients and providers to understand the client’s experiences and his/her sense of self and others and foster the client’s ability to pursue an individualized, self-directed definition of recovery [10]. MERIT is most frequently employed with individuals with psychotic disorders (though the approach itself is transdiagnostic) and is associated with decreases in general distress [11], decreases in positive [12] and negative [13] symptoms of psychosis, and increases in subjective experience of recovery [14]. MERIT has also been found to be effective in improving metacognition, psychosocial functioning, coping with trauma symptoms, co-occurring substance use, and grappling with identity [11,15,16,17,18,19]. These objective measures are corroborated by clients’ subjective experiences in qualitative studies, in which clients reported noticing how MERIT required significant active involvement on their part, and that this active participation felt related to improvements in understanding their own cognitions and the overall effectiveness of the intervention [20].

Although MERIT was initially developed as an individual therapy, it has recently been successfully adapted for group settings (MERITg; [21,22]. In brief, MERITg follows the same principles and tenets as MERIT but uses a written narrative to generate an autobiographical account from each group member simultaneously. Preliminary research suggests that MERITg is feasible and that individuals have found MERITg to be broadly interesting and acceptable [22]. Group interventions are a potentially cost-effective way to increase individuals’ access to mental health care and are especially prevalent in inpatient or intensive outpatient programs, which are highly utilized by individuals with SMI diagnoses [23]. A recent meta-analysis found that many different types of group therapy are broadly effective in treating schizophrenia with small-to-moderate effect sizes [24]. In addition, there is precedence for group interventions targeting the capacity to monitor one’s own thinking in support of implementing cognitive strategies, particularly for individuals with SMIs. Such interventions have been found to lead to improvements in overall symptoms, insight, and functioning [25,26]. 

The group format of MERITg is especially well-suited to meet the goals of MERIT therapy and recovery-oriented care more broadly. While metacognition is an internal, unique experience, it necessarily occurs in the context of relationships and experiences with other people and the outside world. In MERIT, the therapist frequently uses judicious self-disclosure and reflections to elicit awareness of emotions, thoughts, and experiences, and great attention is paid to the interpersonal processes that happen in the therapy room. A facilitator-guided group format allows for increased exposure to others’ experiences and mental states and allows the facilitator and clients to work together in real time to explore dynamic semi-structured interactions (i.e., each group has a specific topic to guide discussion). One of the goals of MERIT is to strengthen metacognitive capacity in order to facilitate decision-making and improve coping. Providing MERIT in a group setting allows individuals to solicit feedback from other group members about potentially challenging situations and to offer their own ideas in turn. This group process, therefore, creates opportunities for increased exposure to potential situations and a supportive environment to practice the application of metacognitive skills. Finally, MERITg helps highlight the importance of peer-led and modeled ideas and goals. As a recovery-oriented practice, MERITg seeks to cultivate clients’ sense of ownership and autonomy, and a group setting decentralizes the role of the facilitator in the intervention. There is therefore a close conceptual alignment between MERITg and the values of the recovery movement; however, to date, there have been no published data examining the association between MERITg participation and recovery-oriented beliefs. Such research is necessary to better understand potential changes that occur in conjunction with participation in the program.

The primary goal of the current study was to address this gap in the literature by exploring the relationship between recovery-oriented beliefs and participation in MERITg in individuals with SMI. In addition, we examined if changes in recovery-oriented beliefs varied depending on the setting (i.e., outpatient or inpatient). We hypothesized that recovery-oriented beliefs would improve after engagement in MERITg for both the outpatient and inpatient groups. 

## 2. Materials and Methods

The present study is a follow-up analysis of a previously published proof-of-concept acceptability and feasibility study of MERITg [22]. The materials and methods of the study were previously published elsewhere [22] and are summarized below.

### 2.1. Recruitment and Procedures

The MERIT psychotherapy group, which was called “Creative Writing”, was offered in two different milieu settings (outpatient and inpatient) as a voluntary, adjunctive treatment to each individual’s treatment plan. The outpatient setting was the VA Connecticut Community Reintegration Program (a part of the Psychosocial Rehabilitation and Recovery Center), which provides a wide range of services to individuals with serious mental illness and substance use disorders. The inpatient setting was a short-term treatment unit for acute psychiatric concerns, including substance use. The group was offered once a week for 13 weeks in the outpatient day program and 6 weeks on the inpatient unit, with varying durations due to staffing constraints. All groups were led by AMSM (at the time, psychology intern) under the clinical supervision of Licensed Clinical Psychologists (MS, JB) and with consultation by PL to ensure MERIT fidelity. The groups were open to any individuals receiving treatment for SMI in the outpatient day program or on the inpatient unit. All participants were fluent in English.

Participants completed a recovery orientation scale at the beginning of their first group (i.e., baseline) and at the end of each group attended (including the first group). Upon the completion of each group, participants were also asked to complete a brief survey about their experience in the group, which is presented elsewhere [22]. Institutional Review Board approval was obtained for chart review and analysis of the qualitative and measurement-based program-evaluation data. 

### 2.2. Group-Based MERIT Intervention

As mentioned previously, the MERITg intervention is described in detail in a prior publication [22], and an intervention guide is now available [21]. In brief, each group was approximately 45 min long, with 15 min for writing and 30 min for discussion. The writing prompt for each session was designed to elicit a narrative account of a specific memory and to be broad enough to apply to most participants (e.g., “In as much detail as possible, describe your first friend or a best friend”; “In as much detail as possible, describe your first job”.). Discussion afterwards centered on both the process of writing and the content of the writing prompt, and participants were invited to share their memory or writing with the group. Facilitators engaged with participants in such a way as to assess metacognitive capacity and moderate interventions accordingly, in line with the eight central tenets of MERIT [10].

### 2.3. Maryland Assessment of Recovery in Serious Mental Illness Scale—Short Form (MARS-12)

Participants’ beliefs about recovery were assessed using the MARS-12 [27]. The MARS-12 is a publicly available short-form questionnaire adapted from the original MARS assessment, which consisted of 25 items [28]. Like the 25-item version, the adapted 12-item version has good internal consistency (Cronbach’s α = 0.92; D. Medoff, personal communication, 1 October 2018). The MARS is based on the Substance Abuse and Mental Health Services Administration definitions of personal recovery [6] and assesses six core themes: self-direction or empowerment, holistic, nonlinear, strengths-based, responsibility, and hope [28]. To assess these concepts, the MARS-12 includes questions such as the following: “I am able to set my own goals in life”; “I feel accepted as who I am”; “I can have a fulfilling and satisfying life”; and “I can make changes in my life even though I have a behavioral health issue”. The MARS-12 uses a 5-point Likert scale ranging from 1 (not at all) to 5 (very much), and the total score is obtained by summing all answers (range 12–60), with a higher score indicating a higher level of recovery-oriented beliefs. 

### 2.4. Statistical Analysis

Statistical analyses were conducted in R version 4.0.2 [29]. Descriptive statistics were conducted to evaluate the characteristics of each group (e.g., total number of unique individuals, average number of individuals that attended each session, number of individuals who attended multiple groups). Quantitative data analysis of the MARS-12 data included paired-samples *t*-tests to examine change in the MARS-12 total score after the last group completed across all subjects, as well as within each location (outpatient and inpatient). Cohen’s *d* was used as an indication of within-subject effect size and was calculated as the mean of the difference between the baseline and last assessment MARS-12 total scores, divided by the standard deviation of that difference. Exploratory bivariate correlations across subjects were used to evaluate the potential dose–response regarding attendance (number of groups attended) and recovery-oriented beliefs (change score on the MARS-12). 

## 3. Results

### 3.1. Participants

Descriptive statistics of the total sample and of the outpatient and inpatient groups separately are presented in Table 1. 

### 3.2. Recovery-Oriented Beliefs: MARS-12

A total of 28 participants completed the MARS-12 at least two times (at baseline and after at least one group), out of a possible 31 participants. One participant from the outpatient group completed a baseline assessment but left the group halfway through and did not complete a post-group assessment. Their baseline data are included, and they were necessarily excluded from change scores and follow-up analyses. Two participants in the inpatient group reported that they did not have any psychiatric illness and that the MARS-12 did not apply to them, and their MARS data are coded as missing. 

#### 3.2.1. Descriptive Statistics 

Descriptive data of the MARS-12 responses are presented in Table 2. MARS-12 scores can range from 12 to 60, with higher scores representing higher levels of recovery-oriented beliefs. Across all participants who completed the MARS-12, scores at baseline and after the final group attended were moderate (M = 45.19, and SD = 9.86; and M = 46.61, and SD = 9.43, respectively) and ranged from 19 to 60. Total MARS-12 scores were lower in the inpatient group compared to the outpatient group at both baseline (M = 41.88, and SD = 13.55; and M = 46.48, and SD = 8.09, respectively) and after the last group attended (M = 42.19, and SD = 15.32; and M = 48.70, and SD = 7.58, respectively). 

In the outpatient group (*n* = 21), the item that received the lowest score on average at baseline was question 2 (“I believe I make good choices in my life”; M = 3.38, and SD = 0.92), and the lowest on average after the final group was question 10 (“I am optimistic that I can solve problems that I will face in the future”; M = 3.80, and SD = 0.83). The item that received the highest score on average at baseline and after the last group was question 12 (“I am responsible for making changes in my life”; M = 4.62, and SD = 0.80; and M = 4.40, and SD = 0.88, respectively).

In the inpatient group (*n* = 8), the item that received the lowest average score at baseline was also question 2 (“I believe I make good choices in my life”; M = 2.75, and SD = 1.39), which is the same item as the outpatient group, and the lowest on average after the final group attended was question 9 (“I can have a fulfilling and satisfying life”; M = 2.75, and SD = 1.58). Similar to the outpatient group, the item with the highest average score at baseline and after the last group completed was also question 12 (“I am responsible for making changes in my life”; M = 4.25, and SD = 1.17; and M = 4.13, SD = 1.13, respectively). 

#### 3.2.2. Change in Recovery-Oriented Beliefs

Contrary to our hypothesis, a statistically significant change in recovery-oriented beliefs was not observed between baseline (at the start of group 1; M = 45.19, and SD = 9.43) and after the last group completed (M = 46.61, and SD = 9.43; *t*(27) = 1.53, *p* = 0.14), and the effect size was small (Cohen’s *d* = 0.29) in the combined outpatient and inpatient sample (*n* = 28). Within the outpatient setting specifically (*n* = 20), recovery beliefs significantly increased (*t*(19) = 2.10, *p* = 0.05) after participation in the group (M = 48.70, and SD = 7.58) compared to baseline (M = 46.48, and SD = 8.09). This change was represented by a moderate effect size (Cohen’s *d* = 0.47). In the inpatient setting (*n* = 8), a significant change in recovery beliefs was not observed (*t*(7) = −0.19, *p* = 0.86) after participation in the group (M = 42.19, and SD = 15.32) compared to baseline (M = 41.81, and SD = 13.55). This change was represented by a very small effect size (Cohen’s *d* = 0.07). 

#### 3.2.3. Effect of Dose on Change in Recovery-Oriented Beliefs

To assess if there was an effect of dose (i.e., number of groups attended) on change in recovery-oriented beliefs, exploratory analyses were conducted. As detailed elsewhere, the average number of groups attended was three (ranging from 1 to 7) and two (ranging from 1 to 5) in the outpatient and inpatient settings, respectively, and approximately half of the participants returned for more than one group (52% in the outpatient group and 50% in the inpatient group; [22]). For the total sample (*n* = 28), change in recovery-oriented beliefs was positively correlated with the total number of groups attended (*r* = 0.45, *p* = 0.02), and this finding was mirrored in the outpatient sample (*n* = 20, *r* = 0.44, *p* = 0.05). The size of the correlation was similar in the inpatient group but did not reach significance (*n* = 8, *r* = 0.47, *p* = 0.24). 

## 4. Discussion

The goal of the present study was to assess if a novel group adaptation of metacognitive therapy (MERITg) was associated with increases in recovery-oriented beliefs in individuals receiving inpatient and outpatient care. Prior proof-of-concept work suggests that this adjunctive intervention is broadly feasible, enjoyable, and acceptable to participants [22], and the current study builds on those initial findings to further probe potential changes in recovery-oriented beliefs that may occur as a result of the metacognitive intervention. Partially consistent with our hypothesis, MERITg was associated with a statistically significant improvement in recovery-oriented beliefs within the outpatient setting, but not on the inpatient unit or across all participants combined (though the effect size was moderate in the combined group; Cohen’s *d* = 0.43). There was also a significant association between MERITg attendance and improvement in recovery-oriented beliefs, such that those who participated in more groups reported greater improvement in recovery-oriented beliefs. While these results are preliminary and based on a small sample, the improvement in recovery-oriented beliefs in those who participated in MERITg in the outpatient setting is particularly encouraging given the relatively brief duration of the group. There is a well-recognized need for effective treatments that are not only delivered efficiently (i.e., in group formats) but that are also feasible from the standpoint of adherence. MERITg may be one such intervention. 

One possible explanation for the lack of improvement in the inpatient group is the smaller sample size (*n* = 10), coupled with the inpatient participants attending fewer sessions overall than the outpatient participants. This theory is supported by the finding that the number of sessions attended was positively correlated with increases in recovery-oriented beliefs across all samples (total, outpatient, and inpatient). The lower attendance rate in the inpatient group is likely due to the typically short duration of inpatient psychiatric hospitalizations and is unlikely to be due to lack of interest or enjoyment, as previously published qualitative data indicated that the group was perceived as positive [22]. Given that the current study was a first-of-its-kind, proof-of-concept trial, a smaller sample size is to be expected, and future work with larger sample sizes and additional measures to assess motivations for participants’ attendance or dropout would be beneficial. 

Another possible explanation for the lack of improvement in the inpatient group could be due to the nature of the inpatient experience. Many inpatient units strive to provide person-centered, recovery-oriented care, but recovery can be difficult to operationalize, and previous work has shown inconsistencies among how different staff members understand and practice recovery-oriented care [30]. In addition, there is an increased emphasis on symptom reduction in inpatient settings compared to outpatient settings, and recovery principles often seek to deemphasize the importance of symptom reduction. An essential part of recovery is the development of personalized values and goals, and the capacity to take ownership and responsibility for those goals. It is possible that the necessarily restrictive nature of the inpatient unit makes it more difficult for individuals, in that moment, to cultivate a sense of autonomy and hope. Consistent with this hypothesis, the mean recovery-oriented belief scores were lower in the inpatient group compared to the outpatient group at baseline (M = 46.15 versus M = 41.81, respectively) and at the end of the intervention (M = 48.70 versus M = 41.38, respectively). However, this is necessarily confounded with symptom severity, which was not measured in the current study but is very likely to have been higher on the inpatient unit than in the outpatient setting. Additional research that explores the relationship between symptom severity, metacognitive capacity, and rates of recovery is warranted. Similarly, measures of recovery-oriented practices across individuals’ treatment would be informative, given that MERITg was offered as an adjunctive treatment in addition to pre-existing treatment plans that often include services such as individual therapy, other psychotherapy groups, medication management, educational and vocational support, and housing assistance. 

Our data provide preliminary support for the impact of MERITg on recovery-oriented beliefs and has several strengths. The development of MERITg is entirely novel and in line with broad therapeutic goals to provide evidence-based, person-centered, recovery-oriented care to individuals with SMI across different therapeutic settings. The inclusion of both outpatient and inpatient settings improves ecological validity and is particularly relevant for individuals with SMI. Similarly, although the participants were considered to have SMI based on their program enrollment, a range of diagnoses were represented across participants, reflecting the heterogeneous nature of individuals receiving inpatient and outpatient treatment. This is also in line with the transdiagnostic nature of both the concept of recovery and metacognitive skill-building. Prior work published from this study [22] used a mixed-methods approach with qualitative and quantitative measures to establish baseline feasibility and acceptability, and the current study builds on that work to elucidate potential mechanisms of change for individuals with SMI. 

Given that the intervention was a novel proof-of-concept study, there are some limitations to consider. First, the number of participants was modest (*n* = 31), and future studies with larger sample sizes, particularly in inpatient settings, would be beneficial. Second, recovery-oriented beliefs were chosen as an important outcome to measure in conjunction with group involvement, and the short-form survey was chosen to reduce participant burden. The short-form MARS-12 has been shown to have good reliability (D. Medoff, personal communication, 1 October 2018); however, future work that compares results across the long- and short-form MARS, over time, and with consideration to the factor structure of each form would be beneficial. In addition to recovery-oriented beliefs, future studies should also examine the impact of MERITg on other clinically relevant outcomes (e.g., objective functioning and level of distress) and other environmental factors that may influence metacognition (e.g., patients’ past or present engagement with other therapeutic interventions that align or conflict with the underlying principles of MERITg, features of patients’ social network, or social interactions). Third, no control group was included, and therefore the results should be interpreted cautiously, and future randomized controlled trials should be conducted. Finally, one study on the relationship between recovery-oriented beliefs and participation in MERITg is not sufficient, and additional research to fully understand this relationship in the future is warranted.

## 5. Conclusions

Metacognition encompasses a complex set of attributes that involve understanding one’s own and others’ thoughts, emotions, beliefs, and unique context in the world. Higher metacognitive ability is associated with better recovery-oriented outcomes for clients experiencing a range of psychiatric diagnoses, and especially for those with serious mental illnesses. MERIT was designed as a one-on-one intervention to foster metacognition and has now been adapted for use in group settings (MERITg), with initial data suggesting feasibility and acceptability. Our preliminary findings support an association between participation in MERITg and improvement in recovery-oriented beliefs, particularly in outpatient settings, with a moderate effect size. A significant increase in recovery-oriented beliefs was not observed in the overall group that included the inpatient unit group, and future work is needed to clarify what effect the therapeutic setting may have on recovery-oriented belief development through MERITg. Our results provide groundwork for better understanding the potential utility of a unique group intervention to support recovery in individuals with serious mental illnesses.

## Figures and Tables

**Table 1:** Descriptive statistics of the sample by setting.

		Total Sample	Outpatient Group ^1^	Inpatient Group
Participant characteristics				
Total *n*		31	21	10
Age	Mean (SD)	49.70 (14.25)	48.90 (13.5)	51.40 (16.9)
	Range	29–72	29–72	30–72
Sex	% male ^2^	66.7%	70.0%	60.0%
	(Number male/female)	(20:10)	(14:6)	(6:4)
Race	% White ^2^	66.7%	60.0%	80.0%
	(B/W/A/NHOPI/AIAN)	(5/20/2/1/2)	(4/12/2/1/1)	(1/8/0/0/1)
Ethnicity	% not Hispanic or Latinx ^2^	83.3%	80.0%	90.0%
	(HL/NHL/U/D)	(3/25/1/1)	(2/16/1/1)	(1/9/0/0)
Education (years)	Mean (SD)	13.03 (1.59)	12.95 (1.61)	13.20 (1.62)
	Range	12–18	12–18	12–16
Psychiatric diagnoses (primary)	% SMI	77.4%	75.0%	100.0%
	(P/BD/MDD/PTSD/GAD)	(6/9/9/6/1)	(2/5/7/6/1)	(4/4/2/0/0)
Comorbid psychiatric diagnoses ^3^	PTSD/GAD/SUD	12/8/1	7/7/1	5/1/1
Group characteristics				
Total *n* of groups offered	--	19	13	6
Average *n* of participants per group	Mean	--	4	3
	(Range)		(1–9)	(1–5)

Abbreviations: *n* = number of participants; SD = standard deviation; B = Black; W = White; A = Asian; NHOPI = Native Hawaiian or other Pacific Islander; AIAN = American Indian or Alaska Native; HL = Hispanic or Latinx; NHL = not Hispanic or Latinx; U = unknown to veteran; D = veteran declined to answer; SMI = serious mental illness, which includes psychotic disorders (schizophrenia, schizoaffective disorder, or psychosis not otherwise specified), bipolar disorder, and major depressive disorder; P = psychotic disorder; BD = bipolar disorder; MDD = major depressive disorder; PTSD = post-traumatic stress disorder; GAD = generalized anxiety disorder; SUD = substance-use disorder. Note: This table reproduces some data previously presented in [21] (see primary text for details). ^1^ One outpatient participant is missing demographic information. ^2^ Percentages reported are based on whichever group is largest within the current sample. ^3^ Participants had up to four comorbid psychiatric diagnoses, with the majority of individuals having at least one comorbid diagnosis (67.7%). Given that individuals could have more than one comorbid diagnosis, counts of diagnoses only and not percentages of a total are provided.

**Table 2:** Summary of recovery-oriented beliefs at baseline and end of treatment by group setting.

	Total Sample ^1^		Inpatient Setting		Outpatient Setting	
	Baseline	End of Treatment ^3^	Baseline	End of Treatment ^3^	Baseline	End of Treatment ^3^
	Mean (SD)	Mean (SD)	Mean (SD)	Mean (SD)	Mean (SD) ^2^	Mean (SD)
MARS-12 item						
1. I am hopeful about the future	3.93 (1.13)	3.91 (1.16)	3.38 (1.41)	3.50 (1.41)	4.13 (0.96)	4.15 (1.10)
2. I believe I make good choices in my life	3.21 (1.08)	3.61 (1.03)	2.75 (1.39)	3.25 (1.28)	3.38 (0.92)	3.90 (0.79)
3. I am able to set my own goals in life	3.90 (0.94)	4.05 (1.06)	4.13 (0.99)	3.81 (1.46)	3.81 (0.93)	4.10 (0.97)
4. When I have a relapse, I am sure that I can get back on track	3.71 (1.31)	3.80 (1.16)	3.56 (1.76)	3.56 (1.76)	3.71 (1.06)	4.00 (0.86)
5. I am confident that I can make positive changes in my life	3.90 (1.14)	3.91 (1.02)	3.38 (1.41)	3.31 (1.39)	4.10 (1.00)	4.10 (0.85)
6. I feel accepted as who I am	3.72 (1.10)	3.98 (1.22)	3.75 (1.28)	4.00 (1.60)	3.71 (1.05)	4.10 (1.07)
7. I believe that I am a strong person	3.97 (1.21)	4.27 (1.06)	3.75 (1.75)	3.94 (1.70)	4.05 (0.97)	4.30 (0.98)
8. I feel good about myself even when others look down on my illness	3.54 (1.32) ^4^	3.76 (1.48)	3.13 (1.46)	3.06 (1.52)	3.70 (1.26) ^4^	3.84 (1.26) ^4^
9. I can have a fulfilling and satisfying life	3.41 (1.18)	3.66 (1.16)	3.13 (1.46)	2.75 (1.58)	3.52 (1.08)	4.05 (0.94)
10. I am optimistic that I can solve problems that I will face in the future	3.64 (1.04)	3.70 (0.90)	3.31 (1.28)	3.19 (1.13)	3.76 (0.94)	3.80 (0.83)
11. I can make changes in my life even though I have a behavioral health issue	3.88 (1.08)	3.80 (1.12)	3.31 (1.16)	3.69 (1.39)	4.10 (1.00)	4.15 (0.81)
12. I am responsible for making changes in my life	4.52 (0.91)	4.39 (0.92)	4.25 (1.17)	4.13 (1.13)	4.62 (0.80)	4.40 (0.88)
Total MARS-12 score	45.19 (9.86)	46.61 (9.43)	41.81 (13.55)	42.19 (15.32)	46.48 (8.09)	48.70 (7.58)
Total *n*	*n* = 29 ^2^	*n* = 28	*n* = 8	*n* = 8	*n* = 21 ^2^	*n* = 20

Note: The Maryland Assessment of Recovery Scale, short form (MARS-12), is in the public domain [28]. Each item is rated on a Likert scale from 1 (not at all) to 5 (very much), with the total score ranging from 12 to 60 and higher scores indicating a higher level of recovery-oriented beliefs. Abbreviations: *n* = total number of participants; SD = standard deviation. ^1^ One veteran participated in both the inpatient and outpatient groups. ^2^ One veteran in the outpatient group completed a baseline assessment but not any post-group assessments. ^3^ Participants completed a varying number of groups. “End of Treatment” refers to the MARS-12 score obtained at the last group completed by the participant. ^4^ One veteran left this item blank but for the words “Not Applicable”.

## Data Availability

Data are contained within the article.
